# Prevalence, Onset, and Course of Suicidal Behavior Among Adolescents and Young Adults in Germany

**DOI:** 10.1001/jamanetworkopen.2019.14386

**Published:** 2019-10-30

**Authors:** Catharina Voss, Theresa M. Ollmann, Marcel Miché, John Venz, Jana Hoyer, Lars Pieper, Michael Höfler, Katja Beesdo-Baum

**Affiliations:** 1 Behavioral Epidemiology, Technische Universität Dresden, Dresden, Germany; 2 Institute of Clinical Psychology and Psychotherapy, Technische Universität Dresden, Dresden, Germany; 3 Division of Clinical Psychology and Epidemiology, Department of Psychology, University of Basel, Basel, Switzerland; 4 Center for Clinical Epidemiology and Longitudinal Studies, Technische Universität Dresden, Dresden, Germany

## Abstract

**Importance:**

Suicidal behavior is a leading cause of death among adolescents and young adults. In light of the ideation-to-action framework, the delineation of frequency and temporal characteristics of such behavior during this developmental period is crucial.

**Objectives:**

To provide lifetime and 12-month prevalence estimates of suicidal behavior, including ideation, plan, and attempt, in adolescents and young adults of the general population, and to provide information about age at onset, temporal characteristics of suicidal behavior, including duration (number of years between onset and last occurrence) and frequency (number of episodes), and transition patterns across suicidal behaviors.

**Design, Setting, and Participants:**

A cross-sectional epidemiological study was conducted in a random community sample of 1180 adolescents and young adults aged 14 to 21 years assessed in 2015 to 2016 in Dresden, Germany. Data analysis was performed from October 2018 to March 2019.

**Main Outcomes and Measures:**

Lifetime and 12-month suicidal behavior (ideation, plan, and attempt) were assessed with a standardized diagnostic interview (Munich-Composite International Diagnostic Interview) by trained clinical interviewers. The onset, frequency, and duration of suicidal behavior were assessed by questionnaire.

**Results:**

Of the 1180 participants (495 male [weighted percentage, 51.7%]; mean [SD] age, 17.9 [2.3] years), 130 participants (10.7%; 95% CI, 9.0%-12.8%), 65 participants (5.0%; 95% CI, 3.9%-6.5%), and 41 participants (3.4%; 95% CI, 2.4%-4.7%) reported lifetime suicidal ideation, plan, and attempt, respectively. Any lifetime suicidal behavior was reported by 138 participants (11.5%; 95% CI, 9.7%-13.7%). Age-specific cumulative incidence estimates indicated an increase in suicidal behavior during adolescence, starting at age 10 years (<1%), increasing slightly until the age of 12 years (2.2%), and then increasing sharply thereafter until age 20 years (13.5%). There were different patterns among female and male participants for ideation, plan, and attempt, with an overall higher incidence among female participants for ideation (hazard ratio, 1.51; 95% CI, 1.02-2.22; *P* = .04), for plan (hazard ratio, 3.31; 95% CI, 1.72-6.36; *P *< .001), and, among those older than 14 years, for attempt (hazard ratio, 3.07; 95% CI, 1.11-8.49; *P *= .03). Of those with suicidal ideation, 66.0% reported persistent or recurrent ideation over more than 1 year with 75.0% reporting more than 1 episode. Of the participants with lifetime suicidal ideation, 47.0% reported a suicide plan and 23.9% reported a suicide attempt. The transition to suicide plan or attempt occurred mainly in the year of onset of suicidal ideation or plan; of those who transitioned, 74.9% transitioned from ideation to plan, 71.2% transitioned from ideation to attempt, and 85.4% transitioned from plan to attempt in the same year.

**Conclusions and Relevance:**

There is an urgent public health need for timely identification of suicidal behavior in adolescents and young adults to terminate persistent or recurrent suicidal tendencies and to interrupt the ideation-to-action transition.

## Key Points

**Question:**

How frequently and when do suicidal ideation, plan, and attempt emerge in adolescence and young adulthood?

**Findings:**

In a cross-sectional, epidemiological study of 1180 adolescents and young adults from a random community sample in Germany, 10.7%, 5.0%, and 3.4% of participants reported lifetime suicidal ideation, plan, and attempt, respectively. The cumulative incidence of suicidal behavior increased after age 10 years more strongly among female than male participants, and the transition from ideation to action occurred mostly during the same year.

**Meaning:**

Adolescence and young adulthood are critical developmental periods for the onset of suicidal behavior, with a short time for targeted interventions to disrupt the ideation-to-action pathway.

## Teaser

This cross-sectional epidemiological study of a random community sample of adolescents and young adults in Dresden, Germany, examines the prevalence and timing of suicidal ideation, planning, and attempt.

## Introduction

Adolescence and young adulthood are critical times for the onset of suicidal behavior.^1,2,3,4,5,6^ Prevalence estimates vary widely across different countries and studies.^1,3^ For adolescents and young adults, lifetime prevalence estimates range from 12.1% to 37.9% for suicidal ideation, 3.0% to 20.3% for plan, and 1.5% to 12.1% for attempt.^1,4,7,8,9,10^

Previous research^11,12,13^ demonstrated that types of suicidal behavior predict each other over time, whereby a gradual pathway from ideation to a plan to an attempt is assumed and is also integrated in different theories and frameworks. In addition to the suggested use of advanced multivariate approaches to improve prediction (eg, machine learning techniques),^14,15,16,17^ a thorough descriptive temporal characterization of suicidal behavior is necessary to improve the identification of critical pathways.

Considering the ideation-to-action transition,^11,18^ cross-national research in adult samples illustrated that approximately one-third of those with suicide ideation continue to make a plan, and less than 30% make an attempt.^3^ The transition from ideation to plan or attempt occurs often during the first year of onset of ideation.^1,19^ There is mixed evidence on whether the age at onset and duration or persistence of ideation or plan increases the risk for transition to more severe types of suicidal behavior.^3,19,20,21,22,23,24^

Only a few epidemiological studies^4,25^ have examined the temporal characteristics of suicidal ideation, plan, and attempt, such as onset, duration, frequency, and transition patterns, in adolescence and young adulthood. To our knowledge, only 1 study^4^ has examined the distribution of age at onset and speed of transition in adolescents, pointing to very limited information about the pathways of suicidal behavior in this critical age group. Therefore, the goal of the present study is to provide (1) lifetime and 12-month prevalence estimates of suicidal behavior, including ideation, plan, and attempt; (2) information about age at onset; (3) temporal characteristics of suicidal behavior, including duration and frequency; and (4) information about transition across suicidal behaviors among adolescents and young adults from a regional general population sample in Germany.

## Methods

### Study Population

In the cross-sectional epidemiological Behavior and Mind Health (BeMIND) study,^26^ a community-based sample of 1180 adolescents and young adults aged 14 to 21 years from Dresden, Germany, was assessed via face-to-face standardized diagnostic interview by trained clinical interviewers. Participants received €50 as an incentive. Detailed information on sampling and study procedures can be found elsewhere.^26^

The BeMIND study was performed in accordance with the ethical standards of the 1964 Declaration of Helsinki and its later amendments, and the study protocol was approved by the ethics committee of the Technische Universität Dresden, Germany. Individuals were eligible to participate if they gave their written informed assent or consent, including written informed consent of all legal guardians for individuals younger than 18 years. This study follows the Strengthening the Reporting of Observational Studies in Epidemiology (STROBE) reporting guideline.

Briefly, the sample was drawn randomly, stratified by sex and age, from the population registry of the city of Dresden, Germany, in 2015. Individuals were invited via letter (with a maximum of 2 reminder letters). Individuals were excluded if they had insufficient German language skills, were permanently institutionalized, or lived outside Dresden during the field phase. Of the 6321 invited individuals, 14.1% were known to be ineligible. Of the remaining individuals, 1180 participated in the study, resulting in a response (participation) proportion of 21.7% (American Association for Public Opinion Research formula RR1).^27^ Assuming that the proportion of eligible participants in cases with unknown eligibility (ie, the 42.8% of the invited individuals or families who did not respond to the invitation letters) is the same as the proportion of eligible participants among those with known eligibility, the estimated overall response proportion was 24.8% (American Association for Public Opinion Research formula RR3).^27^ The main reasons for nonparticipation were lack of time or interest, failure to contact, and arranging suitable appointments. Participation rates were higher among female participants and those with higher education.^26^

### Outcome Measures

#### Lifetime Suicidal Behavior

Lifetime suicidal behavior was assessed in all participants using an updated version (J.H., C.V., J. Strehle, et al, unpublished data, 2019) of the fully standardized computer-assisted Munich-Composite International Diagnostic Interview^28,29,30,31,32^ with the following questions: “Have you ever thought over a period of days or weeks about killing yourself, ie, to attempt suicide?” (ideation); “Have you ever made a specific plan for killing yourself?” (plan, if ideation); and “Have you ever attempted suicide?” (attempt, all participants).

#### Temporal Characteristics

Participants who reported lifetime suicidal behavior were also assessed by questionnaire about age at onset, age at last occurrence, and frequency (ie, number of episodes of ideation and plan and number of attempts). Suicidal behavior in the past 12 months was considered to be present if the age at last occurrence for ideation, plan, or attempt was reported to have occurred in the past 12 months. Duration of suicidal behavior was defined by the time (in years) between age at onset and age at last occurrence. Time between onset of ideation, plan, and attempt was calculated as the difference between the ages at onset (in years).

### Statistical Analysis

Data analysis was conducted from October 2018 to March 2019. Stata statistical software version 14.2 (StataCorp)^33^ was used. Data were weighted to improve representativeness for sex and age,^26^ but frequencies are reported unweighted. Robust 95% CIs were calculated by using the first-order Taylor-series linearization method.^34^ Tests were done at the 2-sided α = .05 level. Age cohort differences (14-17 vs 18-21 years) and sex differences were examined using logistic regression analyses (odds ratios [ORs] including an *F* test); for count data (frequency and duration), negative binominal regression (incidence rate ratios including an *F* test) was used. Odds ratios were used to examine associations between duration and frequency of ideation or plan and transition.

Because any participant could progress into suicidal behavior after observation and until the highest age observed (21 years), our data are right-censored.^35^ This was addressed by survival analyses with event indicators (eg, ideation = 1, no ideation = 0) and the corresponding exit time (ie, minimum of age at onset and age at assessment). The Kaplan-Meier method was used to estimate age-specific cumulative lifetime incidence rates for any suicidal behavior, ideation, plan, and attempt until the age of 21 years, separately for female and male participants.^36^

Sex differences in cumulative incidence curves were analyzed using Cox regression analyses and quantified with hazard ratios (HRs), stratified for age.^36^ For ideation-to-plan, ideation-to-attempt, and plan-to-attempt transition, the time (eg, from ideation to plan) was defined as the age difference in years between the reported ages at onset. Hence, equal age at onset reports (eg, for ideation and plan) were treated as 0 years between ideation and plan. Age at onset of ideation and plan were used explanatorily as covariates in Cox regression analyses to examine associations with transition stratified for sex and age cohort. The proportional-hazards assumption was tested using Schoenfeld residuals.^37^ Once the proportional-hazards assumption was violated, separate Cox regressions were calculated for 2 age intervals before and after the (observed) interception of the curves. Only years with at least 20 individuals at risk are reported.

Missing values for any of the items assessing suicidal behavior (4 participants) were conservatively coded as no suicidal behavior. Two participants did not report an age at last occurrence for ideation or plan; thus, we conservatively imputed the age at onset. Similarly, 3 participants did not report an age at last occurrence for attempt; thus, the age of the most recent plan was used here as an approximation.

## Results

### Sample Characteristics

Overall, 1180 adolescents and young adults (685 female [48.3%] and 495 male [51.7%] participants) aged 14 to 21 years (mean [SD] age 17.9 [2.3] years) participated in the study; 97.1% reported being German citizens. Most (65.1%) lived with their parents, 12.5% lived alone, 5.4% lived with a partner, and 17.1% lived with other people. Most of the participants (76.4%) reported currently receiving or having received a high school or A-level education, 18.6% reported having a middle-school education (secondary school), and 5.0% reported attending other institutions (eg, private schools) or having a low education level (eg, lower secondary school levels).

### Prevalence

Lifetime suicidal ideation, plan, and attempt were reported by 130 participants (10.7%; 95% CI, 9.0%-12.8%), 65 participants (5.0%; 95% CI, 3.9%-6.5%), and 41 participants (3.4%; 95% CI, 2.4%-4.7%), respectively (Table 1). Any lifetime suicidal behavior was reported by 138 participants (11.5%; 95% CI, 9.7%-13.7%). Lifetime prevalence was significantly higher among female than male participants for suicidal ideation (12.9% [95% CI, 10.5%-15.8%] vs 8.7% [95% CI, 6.3%-11.8%]; OR, 1.6 [95% CI, 1.0-2.4]; *P* = .04) and plan (7.8% [95% CI, 6.0%-10.2%] vs 2.4% [95% CI, 1.3%-4.3%]; OR, 3.4 [95% CI, 1.8-6.7]; *P* < .001) but not attempt (4.5% [95% CI, 3.2%-6.5%] vs 2.3% [95% CI, 1.2%-4.4%]; OR, 2.0 [95% CI, 0.9-4.3]; *P* = .07). There were no significant differences between those aged 14 to 17 years vs those aged 18 to 21 years in terms of ideation (9.0% [95% CI, 7.0%-11.6%] vs 11.9% [95% CI, 9.4%-15.0%]; OR, 1.4 [95% CI, 0.9-2.0]; *P* = .12), plan (4.0% [95% CI, 2.7%-5.7%] vs 5.8% [95% CI, 4.1%-8.0%]; OR, 1.5 [95% CI, 0.9-2.5]; *P* = .14), or attempt (2.5% [95% CI, 1.5%-4.1%] vs 4.0% [95% CI, 2.6%-6.0%]; OR, 1.6 [95% CI, 0.8-3.2]; *P* = .15). In the past 12 months, 54 participants (4.0%; 95% CI, 3.0%-5.3%) reported any suicidal behavior (ideation, 54 participants [4.0%; 95% CI, 3.0%-5.3%]; plan, 24 participants [1.7%; 95% CI, 1.1%-2.5%]; attempt, 10 participants [0.6%; 95% CI, 0.3%-1.2%]). A significant sex difference was found only for plan, indicating a higher rate among female participants (19 participants; 2.5%; 95% CI, 1.6%-3.9%) vs male participants (5 participants; 0.9%; 95% CI, 0.4%-2.1%) (OR, 3.0; 95% CI, 1.1-8.1; *P* = .03).

**Table 1.** Prevalence of Suicidal Behavior in Adolescents and Young Adults

Variable	Participants, No.	Participants, No. (Weighted %) [95% CI, %]^a^			
		Any Suicidal Behavior	Ideation	Plan	Attempt
Lifetime					
Total	1180	138 (11.5) [9.7-13.7]	130 (10.7) [9.0-12.8]	65 (5.0) [3.9-6.5]	41 (3.4) [2.4-4.7]
Sex					
Male	495	45 (9.7) [7.2-13.0]	41 (8.7) [6.3-11.8]	12 (2.4) [1.3-4.3]	10 (2.3) [1.2-4.4]
Female	685	93 (13.5) [11.1-16.4]	89 (12.9) [10.5-15.8]	53 (7.8) [6.0-10.2]	31 (4.5) [3.2-6.5]
OR (95% CI)		1.5 (1.0-2.2)	1.6 (1.0-2.4)	3.4 (1.8-6.7)	2.0 (0.9-4.3)
* P* value		.07	.04	<.001	.07
Age, y					
14-17	635	63 (9.5) [7.4-12.1]	60 (9.0) [7.0-11.6]	28 (4.0) [2.7-5.7]	16 (2.5) [1.5-4.1]
18-21	545	75 (13.0) [10.3-16.2]	70 (11.9) [9.4-15.0]	37 (5.8) [4.1-8.0]	25 (4.0) [2.6-6.0]
OR (95% CI)		1.4 (1.0-2.1)	1.4 (0.9-2.0)	1.5 (0.9-2.5)	1.6 (0.8-3.2)
* P* value		.07	.12	.14	.15
Past 12 mo					
Total	1180	54 (4.0) [3.0-5.3]	54 (4.0) [3.0-5.3]	24 (1.7) [1.1-2.5]	10 (0.6) [0.3-1.2]
Sex					
Male	495	16 (3.1) [1.8-5.1]	16 (3.1) [1.8-5.1]	5 (0.9) [0.4-2.1]	2 (0.3) [0.1-1.4]
Female	685	38 (4.9) [3.6-6.8]	38 (4.9) [3.6-6.8]	19 (2.5) [1.6-3.9]	8 (0.9) [0.5-1.9]
OR (95% CI)		1.6 (0.9-3.1)	1.6 (0.9-3.1)	3.0 (1.1-8.1)	NA
* P* value		.12	.12	.03	
Age, y					
14-17	635	36 (5.3) [3.8-7.4]	36 (5.3) [3.8-7.4]	16 (2.3) [1.4-3.8]	9 (1.3) [0.7-2.6]
18-21	545	18 (3.0) [1.9-4.9]	18 (3.0) [1.9-4.9]	8 (1.2) [0.6-2.4]	1 (0.1) [0.0-1.0]
OR (95% CI)		0.6 (0.3-1.0)	0.6 (0.3-1.0)	0.5 (0.2-1.2)	NA
* P* value		.06	.06	.11	

Abbreviations: NA, not applicable; OR, odds ratio.

^a^ Data are weighted to refer to the age and sex distribution in the general population of individuals aged 14 to 21 years in Dresden, Germany. The numbers of participants are unweighted, and percentages are row percentages. Any suicidal behavior comprises ideation, plan, or attempt. Significant sex and age cohort difference at *P* < .05 were determined by logistic regression analyses.

### Estimated Cumulative Incidence and Age-at-Onset Distribution

**Figure 1.**
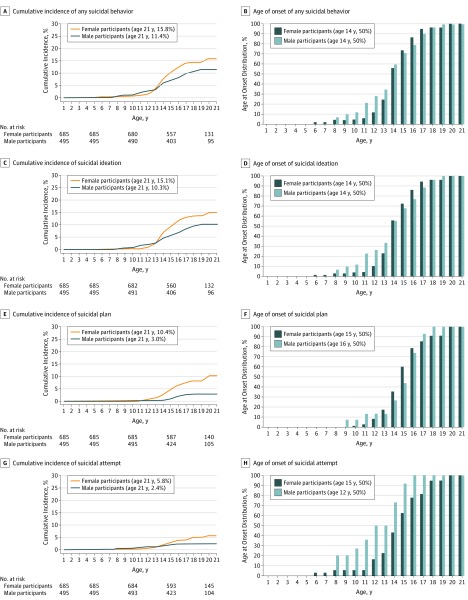
Age-Specific Cumulative Incidence and Age at Onset Distribution for Suicidal Behavior by Sex for 1180 Participants Estimated age-specific cumulative incidence rates from survival analyses and age-at-onset distributions are shown for any suicidal behavior (A and B, respectively), suicidal ideation (C and D, respectively), suicide plan (E and F, respectively), and suicide attempt (G and H, respectively) until age 21 years, stratified by sex and age. Any suicidal behavior comprises any suicidal ideation, plan, and attempt. The median age at onset was determined by the lowest age where the estimated cumulative incidence exceeded one-half of the estimated cumulative incidence at age 21 years. Reported number at risk tables are based on the unweighted Kaplan-Meier estimation.

The estimated cumulative incidence at age 21 years was 13.5% for any suicidal behavior: 12.7% for ideation, 6.6% for plan, and 4.0% for attempt (eFigure in the Supplement). Until the age of 10 years, incidence rates for any suicidal behavior were very low (<1%) and then increased slightly until the age of 12 years (2.2%) and sharply thereafter until age 20 years (13.5%) (Figure 1A and B). There was no significant sex difference in the cumulative incidence of any suicidal behavior (HR, 1.41; 95% CI, 0.97-2.05; *P* = .07). The incidence curves for ideation revealed similar age patterns (Figure 1C and D), but there was an overall higher incidence among female participants (HR, 1.51; 95% CI, 1.02-2.22; *P* = .04). For plan, incidence was rare until age 13 years (<1%), then it increased slightly among male participants and more sharply among female participants until age 18 years (sex difference, HR, 3.31; 95% CI, 1.72-6.36; *P* < .001) (Figure 1E and F). The incidence curve of attempt for female participants stayed low (<1%) until the age of 12 years and then increased slightly linearly until age 18 years (Figure 1G and H). For male participants, the incidence curve of attempt stayed low (<1%) until age 11 years and then increased slightly until age 15 years with no new attempt cases thereafter. The sex difference in cumulative incidence of attempt did not reach statistical significance (HR, 1.97; 95% CI, 0.93-4.17; *P* = .08). However, as the proportional-hazards assumption was violated (ρ = 0.31; *P* = .03), separate Cox regressions by age indicated that the cumulative incidence of attempt was similar among female and male participants before the interception point (age 14 years; HR, 0.88; 95% CI, 0.28-2.81; *P* = .83), but differed thereafter (HR, 3.07; 95% CI, 1.11-8.49; *P* = .03), indicating a higher incidence for female than male participants.

### Duration and Frequency

The present study found both high duration (eg, for ideation, a median of 2 years [range, 1 to 11 years]) and high frequency (eg, for ideation, a median of 3 episodes over days or weeks [range, 1 to >50]) of suicidal behavior (Table 2). Approximately two-thirds of individuals with ideation (66.0%) reported having persistent ideation exceeding 1 year. Approximately one-third of those with plan or attempt reported having a plan (35.9%) or attempt (37.3%) again in at least 1 subsequent year. No significant sex differences in duration were observed (eTable 1 in the Supplement). Three of 4 individuals (75.0%) experienced more than 1 episode of ideation, and half of those with a plan and attempt reported 2 or more events. Female participants reported 27% fewer plan episodes than did male participants if they reported a plan (incidence rate ratio, 0.27; 95% CI, 0.10-0.73; *P* = .01) (eTable 1 in the Supplement).

**Table 2.** Duration and Frequency of Suicidal Behavior Among Those With Ideation, Plan, and Attempt

Variable	Participants, No. (Weighted %)^a^								
	Ideation			Plan			Attempt^b^		
	Total (n = 130)	Female Participants (n = 89)	Male Participants (n = 41)	Total (n = 65)	Female Participants (n = 53)	Male Participants (n = 12)	Total (n = 41)	Female Participants (n = 31)	Male Participants (n = 10)
Suicidal ideation									
Duration, y									
1	43 (34.0)	28 (31.0)	15 (38.2)	18 (28.0)	14 (26.5)	4 (32.8)	11 (38.1)	8 (29.7)	3 (61.8)
2	36 (23.1)	26 (25.2)	10 (20.1)	21 (30.2)	17 (27.4)	4 (38.5)	7 (16.1)	6 (17.8)	1 (11.3)
3	13 (9.4)	10 (11.1)	3 (7.0)	7 (10.1)	6 (11.0)	1 (7.2)	3 (9.4)	3 (12.7)	0
4	13 (12.7)	6 (8.6)	7 (18.5)	6 (10.5)	5 (11.6)	1 (7.2)	3 (10.1)	2 (9.4)	1 (12.1)
5	9 (6.5)	8 (9.8)	1 (2.0)	6 (8.5)	5 (8.9)	1 (7.2)	6 (15.6)	6 (21.1)	0
>5	16 (14.3)	11(14.3)	5 (14.2)	7 (12.8)	6 (14.7)	1 (7.2)	3 (10.8)	2 (9.4)	1 (14.8)
Median (range)	2 (1 to 11)	2 (1 to 9)	2 (1 to 11)	2 (1 to 9)	2 (1 to 9)	2 (1 to 7)	2 (1 to 6)	3 (1 to 6)	1 (1 to 2)
No. of episodes^c^									
1	32 (25.0)	22 (24.8)	10 (25.4)	13 (19.3)	11 (21.3)	2 (13.2)	7 (24.7)	6 (24.5)	1 (25.1)
2	26 (21.7)	16 (18.3)	10 (26.4)	14 (24.2)	10 (18.8)	4 (40.8)	4 (10.0)	4 (13.5)	0
3	19 (12.8)	15 (17.4)	4 (6.4)	10 (14.8)	10 (19.7)	0	5 (15.1)	5 (20.5)	0
4-9	37 (27.9)	27 (30.0)	10 (25.0)	19 (26.5)	17 (30.6)	2 (13.9)	12 (33.8)	9 (32.3)	3(38.2)
>9	16 (12.6)	9 (9.5)	7 (16.9)	9 (15.2)	5 (9.7)	4 (32.1)	5 (16.4)	3 (9.3)	2 (36.7)
Median (range)	3 (1 to >50)	3 (1 to 50)	2 (1 to >50)	3 (1 to >50)	3 (1 to 50)	2 (1 to >50)	3 (1 to >50)	3 (1 to 50)	5 (5 to >50)
Suicide plan									
Duration, y									
1	NA	NA	NA	39 (64.1)	30 (58.6)	9 (80.8)	15 (61.2)	13 (58.2)	2 (76.5)
2	NA	NA	NA	12 (15.2)	10 (16.3)	2 (12.1)	4 (13.5)	3 (11.5)	1 (23.6)
>2	NA	NA	NA	14 (20.7)	13 (25.1)	1 (7.2)	7 (25.4)	7 (30.3)	0
Median (range)	NA	NA	NA	1 (1 to 6)	1 (1 to 6)	1 (1 to 5)	1 (1 to 5)	1 (1 to 5)	1 (1 to 2)
No. of episodes	NA	NA	NA						
1	NA	NA	NA	32 (52.3)	26 (51.7)	6 (54.0)	11 (43.7)	11 (52.2)	0
2	NA	NA	NA	12 (16.6)	12 (22.1)	0	5 (16.3)	5 (19.5)	0
>2	NA	NA	NA	21 (31.1)	15 (26.2)	6 (46.0)	10 (40.0)	7 (28.3)	3 (100)
Median (range)				1 (1 to 40)	1 (1 to 14)	1 (1 to 40)	1 (1 to 40)	1 (1 to 10)	5 (5 to 40)
Suicide attempt									
Duration, y									
1	20 (63.5)	16 (60.2)	4 (73.1)	17 (67.2)	14 (60.8)	3 (100.0)	24 (62.7)	17 (54.4)	7 (78.1)
2	6 (16.4)	6 (22.1)	0	5 (18.8)	5 (22.5)	0	7 (14.8)	6 (19.2)	1 (6.8)
>2	7 (20.1)	5 (17.7)	2 (26.9)	4 (14.0)	4 (16.7)	0	10 (22.5)	8 (26.5)	2 (15.1)
Median (range)	1 (1 to 5)	1 (1 to 4)	1 (1 to 5)	1 (1 to 4)	1 (1 to 4)	1 (1 to 1)	1 (1 to 10)	1 (1 to 10)	1 (1 to 5)
No. of attempts									
1	14 (45.8)	11 (41.4)	3 (58.3)	11 (44.0)	9 (39.1)	2 (69.2)	18 (49.3)	12 (38.1)	6 (69.8)
2	13 (35.9)	12 (43.3)	1 (14.8)	13 (46.9)	12 (50.1)	1 (30.8)	16 (33.9)	14 (44.1)	2 (15.1)
>2	6 (18.3)	4 (15.3)	2 (26.9)	2 (9.1)	2 (10.8)	0	7 (16.8)	5 (17.8)	2 (15.1)
Median (range)	2 (1 to 6)	2 (1 to 6)	1 (1 to 4)	2 (1 to 6)	2 (1 to 6)	1 (1 to 2)	2 (1 to 6)	2 (1 to 6)	1 (1 to 4)

Abbreviation: NA, not applicable.

^a^ Data are weighted to refer to the age and sex distribution in the general population of individuals aged 14 to 21 years in Dresden, Germany. Column percentages are shown with unweighted numbers. Empty cells emerge for suicide plan in those with ideation because the item was assessed only if ideation was presented and therefore is equivalent with the columns for plan (total, 56; female, 53; male, 12).

^b^ Of those with a lifetime suicide attempt, 8 participants (4 female and 4 male) indicated neither ideation nor plan during their life.

^c^ A range of 1 to more than 50 is reported for suicidal ideation because 2 participants indicated more than 50 episodes.

### Transition

Two percent of the sample (26 participants) reported all types of suicidal behavior. Only ideation was reported by 59 participants (5.1%), only plan (if ideation) was reported by 39 participants (3.1%), and only attempt was reported by 8 participants (0.8%). Approximately one-half of those with ideation reported a plan (65 of 130; 47.0%; SE, 4.7%), and nearly one-quarter (33 of 130; 23.9%; SE, 3.9%) reported an attempt; of those who reported an attempt, 23.5% reported an unplanned and 76.5% a planned attempt (Table 3). Overall, 58.1% (26 of 41 participants) of those with an attempt had made a plan, and 24.0% (8 participants) did not report any ideation or plan. Of those with a plan, 38.9% (26 of 65 participants) reported an attempt. Female participants with ideation had a higher probability for plan (OR, 4.0; 95% CI, 1.7-9.4; *P* = .002) and for reporting both plan and attempt (OR, 4.7; 95% CI, 1.2-17.7; *P* = .02) compared with male participants. The number of plan episodes was associated with the occurrence of attempt (OR, per 1 episode, 1.08; 95% CI, 1.00-1.17; *P* = .04). No association was found between duration of ideation or plan as well as frequency of ideation and the ideation-to-action transition (eTable 2 in the Supplement).

**Table 3.** Transition Probabilities for Ideation-to-Action Transitions Among Individuals With Suicidal Ideation Over a Lifetime and the Past 12 Months

Variable	Participants, No.	Participants With Ideation, No. (Weighted %) [95% CI, %]^a^			
		Plan	Attempt	Plan and Attempt	Unplanned Attempt
Lifetime					
Total	130	65 (47.0) [37.8-56.3]	33 (23.9) [17.0-32.5]	26 (18.3) [12.3-26.2]	7 (5.6) [2.5-12.1]
Sex					
Male	41	12 (27.8) [15.5-44.7]	6 (14.8) [6.3-31.0]	3 (7.1) [2.1-21.5]	3 (7.7) [2.2-23.3]
Female	89	53 (60.7) [49.6-70.8]	27 (30.4) [21.3-41.4]	23 (26.3) [17.7-37.2]	4 (4.1) [1.5-10.8]
OR (95% CI)		4.0 (1.7-9.4)	2.5 (0.9-7.1)	4.7 (1.2-17.7)	0.5 (0.1-2.6)
* P* value		.002	.08	.02	.42
Age, y					
14-17	60	28 (43.9) [31.3-57.2]	13 (22.2) [12.9-35.5]	9 (14.3) [7.3-26.2]	4 (7.9) [2.8-20.1]
18-21	70	37 (48.5) [36.2-61.0]	20 (24.8) [15.8-36.6]	17 (20.3) [12.4-31.5]	3 (4.4) [1.3-14.4]
OR (95% CI)		1.2 (0.6-2.5)	1.2 (0.5-2.7)	1.5 (0.6-3.9)	0.5 (0.1-2.8)
* P* value		.61	.74	.37	.46
Past 12 mo					
Total	54	24 (41.6) [28.3-56.2]	10 (15.8) [8.2-28.1]	8 (13.0) [4.6-25.1]	2 (2.8) [0.7-11.2]
Sex					
Male	16	5 (28.0) [10.3-56.8]	2 (11.0) [2.2-40.1]	2 (11.0) [2.2-40.1]	0
Female	38	19 (50.6) [33.9-67.2]	8 (18.9) [9.2-35.0]	6 (14.3) [6.1-29.8]	2 (4.7) [1.1-18.1]
OR (95% CI)		2.6 (0.7-9.8)	1.9 (0.3-11.0)	1.4 (0.2-8.2)	NA
* P* value		.14	.47	.74	
Age, y					
14-17	36	16 (44.0) [27.9-61.5]	9 (24.9) [12.8-43.0]	7 (19.9) [9.2-37.9]	2 (5.1) [1.2-19.5]
18-21	18	8 (38.6) [17.7-64.7]	1 (4.5) [0.5-30.8]	1 (4.5) [0.5-30.8]	0
OR (95% CI)		0.8 (0.2-2.7)	0.1 (0.0-1.3)	0.2 (0.0-1.9)	NA
* P* value		.71	.09	.15	

Abbreviations: NA, not applicable; OR, odds ratio.

^a^ Data are weighted to refer to the age and sex population in the general population of individuals aged 14 to 21 years in Dresden, Germany. Numbers are unweighted and percentages are row percentages. Significant sex and age cohort difference at *P* < .05 were determined using logistic regression analyses. Of those with a lifetime suicide attempt, 8 participants (4 female and 4 male) indicated neither ideation nor plan during their life and were not included in the table.

The observed time for transition ranged from 0 years (ie, the same year) to 7 years for ideation to plan (same year, 50 participants [74.9%]; 1 year, 9 participants [13.9%]; 2-7 years, 6 participants [11.2%]), from 0 to 4 years for ideation to attempt (same year, 23 participants [71.2%]; 1 year, 7 participants [18.7%]; 2-4 years, 3 participants [10.1%]), and 0 to 1 year for plan to attempt (same year, 22 participants [85.4%]; 1 year, 4 participants [14.6%]).

**Figure 2.**
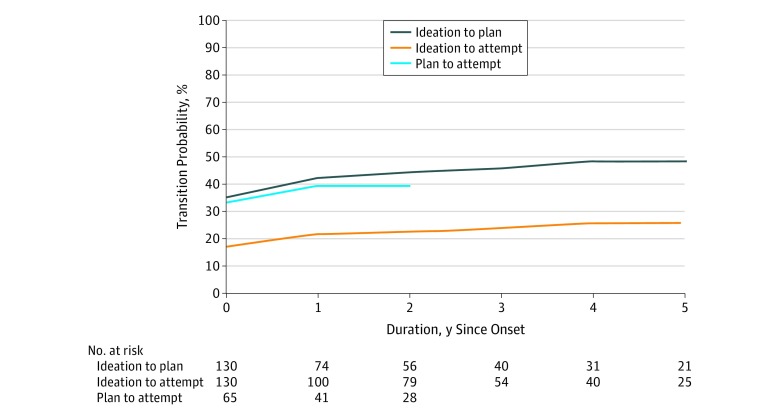
Risk and Speed of Transition From Suicidal Ideation to Plan, Ideation to Attempt, and Plan to Attempt Transition probabilities for suicidal ideation are shown by years since onset of suicidal ideation. The observed ranges were 0 to 7 years from ideation to plan, 0 to 4 years from ideation to attempt, and 0 to 1 year from plan to attempt. Of note, all male participants reported that the first plan and attempt appeared in the same year (3 participants). In the graph, only years with at least 20 individuals at risk are reported. Reported numbers at risk under the graph are based on the unweighted Kaplan-Meier estimation. For the transition from ideation to plan, the probabilities were 35.1% at year 0 (same year) and 48.2% at year 5 (male participants, year 0, 21.8% and year 3, 28.4%; female participants, year 0, 44.8% and year 5, 62.7%; early onset, age <14 years, year 0, 24.9% and year 5, 33.4%; late onset, age >13 years, year 0, 39.0% and year 5, 55.0%). For the transition from ideation to attempt, the probabilities were 17.0% at year 0 and 25.8% at year 5 (male participants, year 0, 10.8% and year 4, 16.1%; female participants, year 0, 21.4% and year 5, 32.7%; early onset, age <14 years, year 0, 16.1% and year 4, 27.5%; late onset, age >13 years, year 0, 17.3% and year 5, 25.4%). For the transition from plan to attempt, the probabilities were 33.2% at year 0 and 39.2% at year 3 (male participants, year 0, 25.6%; female participants, year 0, 35.8% and year 2, 43.5%; early onset, age <15 years, year 0, 34.5% and year 2, 38.5%; late onset, age >14 years, year 0, 32.6% and year 2, 39.5%).

The estimated transition probability was highest in the same year (Figure 2). For the transition from ideation to plan, the probabilities were 35.1% at year 0 (same year) and 48.2% at year 5. For the transition from ideation to attempt, the probabilities were 17.0% at year 0 and 25.8% at year 5. For the transition from plan to attempt, the probabilities were 33.2% at year 0 and 39.2% at year 3. Overall, female participants showed at least a 36% higher risk for the ideation-to-plan transition than male participants (HR, 2.46; 95% CI, 1.36-4.43; *P* = *.*003). No significant sex differences emerged for ideation-to-attempt (HR, 2.14; 95% CI, 0.92-4.95; *P* = .08) and plan-to-attempt (HR, 2.10; 95% CI, 0.62-7.10; *P* = .23) transitions.

Age at onset of ideation was associated with plan (HR, 1.11; 95% CI, 1.01-1.22; *P* = .04); that is, those with a later onset of ideation had overall a higher risk for a plan. No associations were found between age at onset of ideation (HR, 0.95; 95% CI, 0.77-1.16; *P* = .61) and age at onset of plan (HR, 0.97; 95% CI, 0.81-1.15; *P* = .70) in the transition to attempt. No association between age at onset and transition patterns were found.

## Discussion

The key findings of this epidemiological study in a general population sample of participants aged 14 to 21 years from Dresden (Germany) are as follows. First, there was a high lifetime prevalence (11.5%) and estimated cumulative incidence until age 21 years (13.5%) for any suicidal behavior. Second, suicidal behavior typically increased after the age of 10 years and most sharply at age 13 to 14 years, with different onset patterns by sex and for ideation, plan, and attempt. Third, suicidal behavior was frequently persistent and recurrent. Fourth, transition to more serious suicidal behavior was considerable: a plan was reported by 47.0% and an attempt by 23.9% of those with ideation. Approximately 17.0% to 35.1% of participants experienced an ideation-to-action transition within the same year. With these findings, the current study extends the knowledge on suicidal behavior derived by the limited number of previous studies in the critical age span of adolescence.

The prevalence rates in the current study are in the lower range of estimates from previous studies of adolescents around the world,^1,7,10,25,38,39,40,41^ including Germany.^9,10,39,40^ However, our estimates are comparable to those of studies using similar assessment methods (eg, standardized interview) among participants aged 13 to 18 years from the United States^4^ and adults worldwide.^1,3,42^ The lower estimates for suicidal behavior in the present study compared with other studies could be explained by sampling procedures. Previous studies were mostly based on school-based samples that include more individuals with lower education and lower socioeconomic status,^38,39,40^ which seem to be associated with suicidal behavior, although results differ by type of suicidal behavior, sex, and age.^1,43^ In the study by Brunner and colleagues,^39^ a representative sample of 5759 ninth-grade students was studied between 2004 and 2005 in the Rhein-Neckar district in Germany, resulting in higher prevalence estimates (suicidal ideation, 14.4%; plan, 6.5%; attempt, 7.9%). In contrast to previous studies, the current study revealed a female preponderance in prevalence for ideation and plan only, rather than higher rates for female than male participants in general.^1,7,43^ To our knowledge, no consistent findings exist for differences in prevalence by age in adolescents.^20,43^ In the present study, no age cohort differences were found, indicating that suicidal behavior is of relevance throughout adolescence.

The occurrence of suicidal behavior throughout adolescence and young adulthood is characterized by the cumulative incidence and age at onset distribution patterns for suicidal behavior, demonstrating the high-risk period for first onset during adolescence and underlining the need for sensitive assessment throughout development. More specifically, the time from age 12 to 16 years appears to be the first crucial risk phase, with a particularly high incidence at ages 13 to 14 years. Adult samples usually report a considerable higher median age at onset, mostly in the mid-20s, for ideation, plan, and attempt,^1,3,5,44^ likely as a result of both retrospective bias and real episodes in adulthood. The incidence curves in the present study are nearly identical to, although somewhat lower than, the ones presented by Nock and colleagues^4^ for US participants aged 13 to 18 years. Adding to the limited information about sex differences so far,^2,4^ female participants showed a higher incidence of ideation and plan during adolescence compared with male participants and for attempt from age 14 years onward in the present study. The sample size for male participants reporting an attempt was small and no new cases of plan and attempt were observed in male participants after ages 15 and 16 years. Therefore, sex differences have to be interpreted with caution. Available statistics^1,45^ clearly demonstrate a male preponderance for completed suicides, with sex difference emerging in adolescents.

Regarding temporal characteristics of suicidal behavior, so far only a few studies^23,46,47^ are available using self-reports with different time intervals for adult samples. Some studies^20,48^ used data-driven approaches, such as latent-class analyses based on dimensional measures, and found different patterns in girls and boys aged 12 to 15 years.^20^ The present study demonstrates both high duration (eg, for ideation, a median of 2 years) and high frequency (a median of 3 episodes over days or weeks) of suicidal behavior. Sex differences were evident only in terms of a higher number of plan episodes among male participants.

Compared with the results by Nock and colleagues^4^ from the United States, our point estimates were higher for transition probabilities for ideation-to-plan (47.0% [SE, 4.7%] in the present study vs 33.4% [SE, 3.2%] in the study by Nock et al) and lower for ideation-to-attempt (23.9% [SE, 3.9%] in the present study vs 33.9% [SE, 3.7%] in the study by Nock et al). Results regarding the speed of transition are similar to previous studies indicating that the first year after onset is most critical.^3,4^ Female participants showed a higher risk for ideation-to-plan transition compared with male participants, which differs from previous findings based on similar assessments.^3,4^ The current study adds to the literature that a higher number of episodes of planning are associated with attempt. Also, age at onset of ideation seems to be relevant for the ideation-to-plan transition, with a later onset associated with a higher transition risk. With regard to timing of transition, more fine-grained studies are needed to understand the concrete pathways of suicidal behavior (eg, by using ecological momentary assessment).

### Limitations

The present study is one of a few epidemiological studies that examined lifetime suicidal behavior in a population-based sample. Given the focus on adolescents and young adults, all results are restricted up to age 21 years and to a region (major city and capital of Saxony) in the eastern part of Germany. Here, the population density and mean age are similar to those in most larger cities in Germany, with a lower proportion of migrant individuals.^26^ The participation rate was low. However, low participation is a general trend in recent epidemiological studies,^49,50,51^ especially among adolescents,^52,53^ and is not necessarily associated with the validity of the result.^51^ In the current study, participation bias could have had an effect on the prevalence estimates, although rates are similar to those of other studies using a similar design.^1^ Compared with the general population of Dresden, a smaller number of individuals with lower education participated in the study,^26^ which was previously associated with more suicidal behavior, although as pointed out already, the results differed by type of suicidal behavior, sex, and age,^1,43^ and this could have led to an underestimation of prevalence estimates. The sample also differed slightly from the general population in that there were more female participants. Sample weights were used to adjust for sex and age differences. The limited sample size in the current study is associated with comparatively high random error, specifically in risk estimates close to the age of 21 years and transition probabilities. Previous studies^54,55^ indicated that suicidal behavior is more common among nonresponders, so there might be an underestimation of the prevalence in the current study. Moreover, as with other epidemiological studies,^54^ the current study did not assess currently institutionalized individuals (eg, hospitalized), who might have a higher risk for suicidal behavior. Hence, we assume that suicidal behavior is even more prevalent in the general population than our results suggest. In contrast, nonresponder analyses for the present study indicated that participation tended to be higher among those with more mental health problems,^26^ which could be associated with reported suicidal behavior. The presence of a mental disorder (eg, depression or substance use disorder) during an interview about suicidal behavior could have had an effect on the recall of suicidal episodes, although so far little is known about the direction of this effect across different psychopathologic disorders.

Information assessed in the study including age at onset reports was based on retrospective reports, which might be affected by recall bias.^32,56,57,58^ On the one hand, reviews^59,60^ found that individuals can recall especially adverse experiences with sufficient accuracy. On the other hand, the test-retest reliability of lifetime suicide attempt seems to be limited, with one-third of cases not reporting the attempt 4 years later^32^ or reporting it inconsistently over time.^54^ Previous studies (J.H., C.V., J. Strehle, unpublished data, 2019)^30^ analyzing the reliability of the Composite International Diagnostic Interview time-related items, however, found generally high test-retest reliability for age at onset reports. In addition, face-to-face assessment of suicidal behavior might have resulted in fewer reports of suicidal behavior^7,61^ and, thus, in underestimated prevalence or cumulative incidence. For duration and frequency, observed data were used and censoring was not taken into account, which might have led to an underestimation of duration and frequency. By defining duration as the number of years between onset and last occurrence, it remains unknown whether suicidal behavior persisted throughout the years or rather showed a recurrent pattern with years of remission in between, which would have led to an overestimation of duration.

## Conclusions

Adolescence and young adulthood are critical developmental periods for the onset of suicidal behavior, with a small time frame for recognition and targeted interventions to disrupt the ideation-to-action pathway. The present study implicates, on the one hand, the need for immediate short-term interventions to prevent escalating suicidal behavior, ideally around ages 12 to 16 years, and, on the other hand, longer-term monitoring and possibly interventions to prevent or diminish the impact of persistent or recurrent patterns of suicidal behaviors across adolescence. Mobile technologies^62^ may be promising new tools for recognition and intervention.

## Notes

Supplement. **eFigure.** Age-Specific Cumulative Incidence for Suicidal Behavior **eTable 1.** Sex Difference in Duration and Number of Episodes or Attempts **eTable 2.** Association Between the Ideation-to-Action Transition and Duration and Number of Episodes or Attempts
