# Supplementary material for: Prevalence, Onset, and Course of Suicidal Behavior Among Adolescents and Young Adults in Germany
Source: JAMA Netw Open. 2019 Oct 30;2(10):e1914386. doi: 10.1001/jamanetworkopen.2019.14386 (PMC6824228; doi:10.1001/jamanetworkopen.2019.14386)
Supplement: Supplement. — eFigure. Age-Specific Cumulative Incidence for Suicidal Behavior eTable 1. Sex Difference in Duration and Number of Episodes or Attempts eTable 2. Association Between the Ideation-to-Action Transition and Duration and Number of Episodes or Attempts [file jamanetwopen-2-e1914386-s001.pdf]

Supplementary Online Content

Voss C, Ollmann TM, Miché M, et al. Prevalence, onset, and course of suicidal behavior among adolescents and young adults in Germany. *JAMA Netw Open*. 2019;2(10):e1914386. doi:10.1001/jamanetworkopen.2019.14386

eFigure. Age-Specific Cumulative Incidence for Suicidal Behavior

eTable 1. Sex Difference in Duration and Number of Episodes or Attempts

eTable 2. Association Between the Ideation-to-Action Transition and Duration and Number of Episodes or Attempts

This supplementary material has been provided by the authors to give readers additional information about their work.

© 2019 Voss C et al. *JAMA Network Open.*

eFigure. Age-specific Cumulative Incidence for Suicidal Behavior.

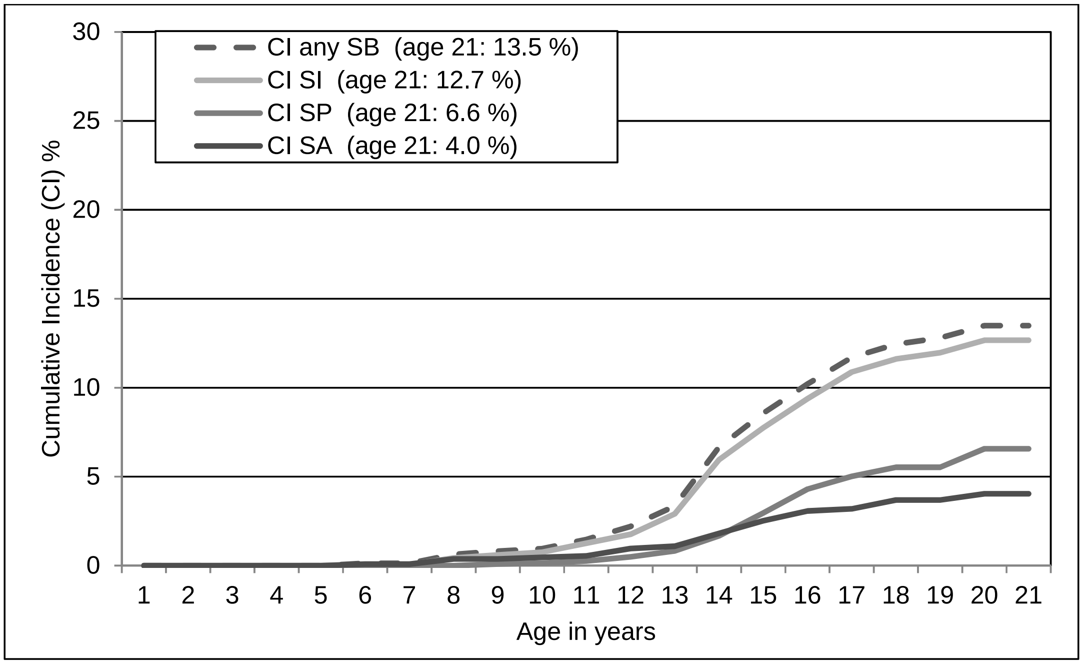

Estimated age-specific cumulative incidence for suicidal behavior are shown until age 21 (N=1180) stratified for age. Any suicidal behavior (SB) comprises any suicidal ideation (SI), plan (SP) and attempt (SA).

© 2019 Voss C et al. *JAMA Network Open.*

eTable 1. Sex Difference in Duration and Number of Episodes or Attempts.

	Sex difference[a]		
	**IRR**	**95%**	**P**
Suicidal ideation (N=128)			
Duration in years	0.98	0.70-1.38	.92
No. of episodes	1.06	0.68-1.67	.80
Suicide plan (N=65)			
Duration in years	1.31	0.83-2.07	.25
No. of episodes	0.27	0.10-0.73	.01
Suicide attempt (N=41)			
Duration in years	1.30	0.74-2.30	.36
No. of attempts	1.29	0.83-2.01	.25

Abbreviation: NA, is not applicable; IRR, incidence rate ratios.
[a]Data are weighted to refer to the age and sex population in the general population of aged 14 to 21 years in Dresden. The number of participants are unweighted. Significant sex difference at p < .05 were determined using negative binominal regression regression analyses. Two participants were excluded from the analyses for number of episodes of suicidal ideation because they reported 400 and more episodes.

© 2019 Voss C et al. *JAMA Network Open.*

eTable 2. Association Between the Ideation-to-Action Transition and Duration and Number of Episodes or Attempts.

	Transition to Plan[a]			Transition to Attempt[a]		
	**OR**	**95%**	**P**	**OR**	**95%**	**P**
Suicidal ideation (N=128)						
Duration in years	0.99	0.83-1.17	.88	1.01	0.81-1.26	.94
No. of episodes	1.04	0.97-1.11	.31	1.05	0.99-1.11	.12
Suicide plan (N=65)						
Duration in years	NA			0.98	0.64-1.51	.94
No. of episodes	NA			1.08	1.00-1.17	.04

Abbreviation: NA, is not applicable; OR, odds ratio.
[a]Data are weighted to refer to the age and sex population in the general population of aged 14 to 21 years in Dresden. The number of participants are unweighted. The associations between the ideation-to-action transition and duration in years and number of episodes or attempts were determined by logistic regression analyses. Two participants were excluded from the analyses for number of episodes of suicidal ideation because they reported 400 and more episodes.

© 2019 Voss C et al. *JAMA Network Open.*
